# A study protocol for a predictive model to assess population‑based risk of adverse pregnancy outcomes: The Adverse Pregnancy Outcomes Population Risk Tool (PregPoRT)

**DOI:** 10.1186/s41512-026-00220-3

**Published:** 2026-03-03

**Authors:** Sabrina Chiodo, Sonia M. Grandi, Jessica Gronsbell, Laura C. Rosella

**Affiliations:** 1 Division of Epidemiology, Dalla Lana School of Public Health, University of Toronto, 155 College St., Toronto, ON Canada; 2 Child Health Evaluative Sciences, The Hospital for Sick Children, Toronto, ON Canada; 3 Faculty of Arts & Science, Department of Statistical Sciences, University of Toronto, Toronto, ON Canada; 4 ICES, Toronto, ON Canada; 5 Institute for Better Health, Trillium Health Partners, Mississauga, ON Canada; 6 Laboratory Medicine and Pathobiology, Temerty Faculty of Medicine, University of Toronto, Toronto, ON Canada

**Keywords:** Adverse pregnancy outcomes, Risk prediction model, Maternal health, Population health, Social determinants of health, Health equity, Public health planning, Prediction modeling

## Abstract

**Background:**

Adverse pregnancy outcomes (APOs), such as gestational diabetes, preeclampsia, and placental abruption, are major contributors to maternal and fetal morbidity and mortality, with implications for individual long-term health and health system performance. Existing prediction models for APOs rely primarily on clinical or biomarker data, with few incorporating social, behavioral, or environmental determinants that are critical for shaping perinatal outcomes. This study describes the development and validation protocol for the Adverse Pregnancy Outcomes Population Risk Tool (PregPoRT), a novel, population-based prediction model designed to estimate APO risk using population-based and routinely collected survey and administrative data in Canada.

**Methods:**

PregPoRT will be developed using a retrospective cohort of female-identifying individuals, aged 15–49, who participated in the Canadian Community Health Survey (CCHS) between 2000 and 2017, and had a subsequent delivery hospitalization within two years recorded in the Discharge Abstract Database (DAD). Pre-pregnancy predictors were selected according to a health equity-informed framework by Kramer and colleagues (2019), and include biomedical, behavioral, social, and environmental variables from the CCHS, the Canadian Marginalization Index (CAN-Marg), the Canadian Urban Environmental Health Research Consortium (CANUE), and the Canadian Active Living Environments (Can-ALE) dataset. The primary outcome is a composite measure of APOs (gestational diabetes, preeclampsia, or placental abruption), identified using validated ICD codes. A Weibull accelerated failure time model will be used to estimate the risk of experiencing an APO. Continuous variables will be modeled with restricted cubic splines. Variable selection will be performed using the Least Absolute Shrinkage and Selection Operator (LASSO), and model performance will be assessed via discrimination, calibration, and overall accuracy. Validation strategies include split-sample, bootstrap, and temporal validation using later CCHS cycles. Survey weights will be applied throughout to ensure national representativeness.

**Discussion:**

PregPoRT will be the first Canadian prediction model for APOs that leverages nationally representative, linked survey and administrative data and explicitly integrates social, behavioral, and environmental determinants of health, domains that have been largely absent from prior models. By incorporating modifiable and socially patterned risk factors, the tool is designed to support public health planning, resource allocation, and maternal health equity monitoring.

## Introduction

Adverse pregnancy outcomes (APOs), such as gestational diabetes, placental abruption, and hypertensive disorders of pregnancy, are leading contributors to maternal and fetal morbidity and mortality, with long-term consequences for individuals, their families, and health systems [1, 2]. For instance, preeclampsia can lead to permanent organ damage, increased stroke risk, and cognitive impairment [3], while gestational diabetes raises the risk of developing type 2 diabetes more than seven-fold, with half of affected individuals progressing to the condition within a decade [4]. In high-income countries, up to a third of pregnancies are affected by at least one APO. In Canada, the burden is not only clinical but economic; preeclampsia accounts for over $3 million CAD annually to healthcare costs [5]. Despite this, prevention efforts are hampered by gaps in identifying high-risk subgroups and aligning resources accordingly. Addressing APOs requires a shift from a purely clinical focus to a broader population health approach that incorporates the social and structural factors shaping maternal health.

Disparities in APOs are well documented, with higher risks among individuals from lower socioeconomic backgrounds, racialized communities, and underserved geographic areas [6–12]. In 2018, the American College of Obstetricians and Gynecologists (ACOG) emphasized the importance of considering social determinants, such as income, education, and access to care, in reproductive health [13]. Building on this guidance, Kramer and colleagues (2019) proposed a health equity–oriented framework that integrates both proximal biomedical and behavioral factors (e.g., chronic conditions, alcohol use, smoking) and distal social and structural determinants (e.g., social support, neighborhood environments, systemic racism) in shaping pregnancy outcomes [14]. These interacting layers of influence underscore the need for predictive models that reflect the lived realities of pregnant individuals and the structural contexts in which they navigate pregnancy.

Population-based risk prediction tools present a valuable opportunity to close existing gaps by identifying at-risk groups using routinely collected, population-level data. However, few current models for APOs account for social or environmental determinants, despite their well-established influence on outcomes. Currently, only two prediction models integrating social determinants of health exist—one for the Medicaid population in the US [15] and another focused on fetal APOs (low birth weight and preterm birth) [16]. Neither is adapted for the Canadian context, and both overlook environmental determinants, which are increasingly important due to climate change [17]. For an effective population algorithm, input variables must be representative of the entire population (i.e., population-based), relevant to health policy, routinely collected, and regularly updated [18]. The omission of self-reported survey data means these models often overlook critical sociodemographic characteristics (e.g., immigration status, ethnicity), socioeconomic indicators (e.g., income, education), and health behaviors (e.g., smoking, alcohol use). Incorporating these variables from administrative health records and survey sources could enhance predictive accuracy and yield more nuanced, equity-focused population health forecasts.

In Canada, the infrastructure to build a population-based, equity-informed prediction model already exists. The Canadian Community Health Survey (CCHS) [19] is designed to be representative of the Canadian population and collects rich data on social, behavioral, and contextual factors and can be linked to administrative hospital records through the Discharge Abstract Database (DAD) [20]. When further linked with area-level and environmental datasets such as the Canadian Marginalization Index (CAN-Marg) [21], the Canadian Urban Environmental Health Research Consortium (CANUE) [22], and the Canadian Active Living Environments Index (Can-ALE) [23], this data ecosystem provides a unique foundation for population health surveillance and planning.

To capitalize on this opportunity, we propose the development and validation of the Adverse Pregnancy Outcomes Population Risk Tool (PregPoRT), a novel, population-based algorithm designed to predict the risk of maternal APOs using linked survey, administrative, and environmental data. PregPoRT will estimate the two-year risk of experiencing a composite outcome of gestational diabetes, preeclampsia, or placental abruption, following participation in the CCHS. Informed by the Kramer and colleagues (2019) framework [14], PregPoRT will integrate biomedical, behavioural, social, and environmental predictors to generate actionable insights for public health professionals, healthcare planners, and researchers.

This study protocol outlines the data sources, cohort structure, model development and validation strategy, and performance assessment plan. PregPoRT will be developed in accordance with best practices for prognostic modeling, guided by the Transparent Reporting of a multivariable prediction model for Individual Prognosis or Diagnosis (TRIPOD) statement [24] and statistical recommendations from Harrell and Steyerberg [25, 26]. This protocol is designed to ensure methodological transparency, minimize the risk of overfitting, and facilitate the responsible application of PregPoRT in public health decision-making and maternal health equity initiatives.

## Methods

### Data sources

The cohort was established through a multi-step linkage process combining population-based survey data with hospital administrative records and environmental/contextual datasets. Using Statistics Canada’s Social Data Linkage Environment (SDLE) at the University of Toronto Research Data Centre [27], CCHS respondents were individually linked to delivery records in the DAD. Area-level and environmental exposures were then appended using participants’ residential postal codes reported at the time of their CCHS interview.

### Primary data sources

#### Canadian community health survey (CCHS)

The CCHS is a nationally representative cross-sectional survey conducted by Statistics Canada that collects information on health status, healthcare use, and social determinants of health [28]. Initially administered biennially, it transitioned to an annual format in 2007, with approximately 65,000 individuals sampled each year across all ten provinces and three territories through a multistage, stratified design. In recent years, targeted sampling strategies have been introduced to enhance representation of equity-deserving populations. Certain groups are not included in the survey frame—specifically, individuals living on reserves, full-time members of the Canadian Forces, institutionalized populations, and residents of some remote regions. While these groups represent less than 3% of the Canadian population, they are important populations whose health experiences are not captured by the CCHS.

The survey includes a stable set of core content (e.g., chronic disease, access to care) alongside rotating thematic modules (e.g., mental health, food insecurity), yielding individual-level measures spanning sociodemographics (age, sex, racialized group, immigration status, education, income) and health behaviours (tobacco use, alcohol, physical activity, diet). It is administered in English and French via computer-assisted telephone interviews, in-person interviews, and a secure web questionnaire.

For this protocol, the CCHS contributes preconception exposures, supports equity-focused subgroup analyses, and provides behavioural and contextual covariates that are typically unavailable in clinical or administrative data.

#### Discharge Abstract Database (DAD)

The DAD, maintained by the Canadian Institute for Health Information (CIHI), captures all hospital separations, including discharges, transfers, and in-hospital deaths, from acute care facilities across Canada, excluding Quebec [20]. It includes detailed data on obstetrical deliveries, newborns, and stillbirths. As ~ 98% of Canadian births occur in hospitals, most pregnant CCHS participants can be reliably linked to delivery records in the DAD [29].

Each record includes demographic, administrative, and clinical data abstracted from hospital charts: age, sex, admission/discharge dates, discharge disposition, diagnoses (up to 25), procedures, and length of stay. Diagnoses and interventions are coded using ICD-10-CA and CCI standards since 2004–05 (ICD-9-CA prior). Data undergo rigorous quality checks at both hospital and CIHI levels [30]. In this study, the DAD was used to identify delivery hospitalizations and ascertain APOs, including gestational diabetes, preeclampsia, and placental abruption. Data from Quebec are not included, as hospitalizations in that province are recorded separately and thus not captured in this cohort.

### Secondary linked environmental data sources

#### Canadian Marginalization Index (CAN-Marg)

The Canadian Marginalization Index (CAN-Marg) is an area-based measure of social inequality, originally created by the Centre for Urban Health Solutions at St. Michael’s Hospital and now maintained by Public Health Ontario [21, 31]. It is constructed from Canadian census indicators using principal component analysis to generate standardized scores across four domains of marginalization: material deprivation, residential instability, dependency, and ethnic concentration. Scores are available at the dissemination area (DA) level. Although the index has not been updated beyond 2016, it continues to be widely applied in Canadian population health research as a proxy for neighbourhood-level social determinants of health.

#### Canadian Urban Environmental Health Research Consortium (CANUE)

CANUE provides nationally harmonized, high-resolution environmental exposure data that are linkable to individual-level health and survey data through residential postal codes [22]. CANUE aggregates data on environmental exposures across multiple domains, including air pollution, greenness, and other climate-related factors. CANUE datasets provide spatially precise exposure estimates at a 1 km² resolution or at the postal code level, depending on the variable. Environmental exposures were matched based on the year of the CCHS interview or the closest available year.

#### Canadian Active Living Environments Index (Can-ALE)

Can-ALE is a nationally standardized, area-level index that quantifies the built environment’s supportiveness for active living [23]. Developed by the Geo-Social Determinants of Health Research Group at McGill University with support from the Public Health Agency of Canada, Can-ALE provides objective measures of neighbourhood walkability and access to amenities that facilitate physical activity. These measures are derived from open-source geographic data and are available for all census dissemination areas (DAs) in Canada for the years 2006 and 2016.

### Study design

PregPoRT is based on a population-based retrospective cohort, consisting of individuals identified as female at birth, aged 15–49, who participated in the CCHS between 2000 and 2017 and had a subsequent delivery hospitalization recorded in the DAD within two years of their survey interview. Delivery outcomes (live birth or stillbirth) were identified using ICD-9 codes 650, V27x, V30x–V39x and ICD-10 codes Z37x, Z38x, O80x, and O82x. To avoid outcome heterogeneity, individuals with multifetal pregnancies were excluded, identified using ICD-9 codes 651, V27.2–V27.7, V31–V37, V33–V36 and ICD-10 codes Z37.2–Z37.7, Z38.3–Z38.8. Participants were followed from the date of their CCHS interview until the date of their delivery hospitalization, up to a maximum of two years, to ensure temporal alignment of pre-pregnancy exposures with outcomes and reduce misclassification. This window was also chosen because prior research indicates that social determinants of health remain relatively stable over a two-year period [32].

Cohort creation and preliminary analyses identified 13,360 deliveries, which exceeds the minimum required sample size for model development (see Section E, Sample Size), and an APO prevalence of 7.6%. This ensures an adequate number of outcome events to support stable estimation and reliable performance in predictive modeling.

### Identification of potential predictor variables

The selection of predictors for PregPoRT was guided by the health equity-informed framework for maternal health, developed by Kramer and colleagues [14]. This framework highlights that while clinical factors directly influence APO risk, broader contextual factors, such as social support, healthcare access, and neighbourhood environments, act as upstream regulators of health, shaping behaviours, care utilization, and exposure to stressors. To ensure PregPoRT reflects this multifaceted reality, predictors were selected based on their availability across CCHS cycles and provinces, relevance to maternal health, and support from the existing literature. These variables were then organized along two intersecting axes of the framework: the life course health trajectory (horizontal axis) and multilevel determinants of health (vertical axis).

Along the life course axis, predictors capture preconception exposures, psychosocial stressors, and accumulated health conditions. These include sociodemographic and economic characteristics (e.g., age, household income, education, immigration status, food insecurity, visible minority status, marital status, and employment); health behaviours (e.g., alcohol consumption, cigarette smoking, fruit and vegetable intake, and physical activity); chronic conditions (e.g., pre-pregnancy diabetes, hypertension, asthma, arthritis, migraines, and multimorbidity); psychosocial stress and perception (e.g., life stress, community belonging, life satisfaction, self-rated health, and self-rated mental health); and reproductive and preconception health factors (e.g., body mass index [BMI], parity, folic acid use, and prior APOs such as preterm birth, live birth, or spontaneous abortion).

On the vertical axis, predictors capture broader systemic and environmental influences. These include healthcare access-related factors (e.g., access to a regular doctor, Pap smear screening, flu shot receipt, and mental health consultations); built environment variables from Can-ALE (e.g., urbanicity, intersection and dwelling density, and transit access); environmental exposures from CANUE (e.g., fine particulate matter [PM₂.₅], nitrogen dioxide [NO₂], ozone [O₃], and greenness); and area-level social determinants measured using CAN-Marg indices of neighbourhood deprivation, ethnic concentration, residential instability, and dependency, as well as census-based area-level income quintiles.

A complete list of variables and definitions is provided in Table 1.

**Table 1:** Specification of predictor variables included in PregPoRT, with corresponding degrees of freedom (df), source Dataset, and variable scale

Variable Grouping	Variable	Definition	df	Dataset	Scale
Horizontal Axis: Life Course Social and Health Trajectory Captures preconceptional exposures, stress, and accumulated chronic conditions					
Sociodemographic & Economic Status	Age	Continuous	4	CCHS	Continuous
	Household income	Quintile 1 (lowest 20%) to Quintile 5 (highest 20%)	4	CCHS	Ordinal
	Household size	Continuous	4	CCHS	Continuous
	Education	Less than Secondary; Secondary Graduate; Post-secondary Graduate	2	CCHS	Ordinal
	Immigration status	Canadian; Recent immigrant (< 10 years); Established immigrant (> = 10 years)	2	CCHS	Categorical
	Food insecurity	Food secure; Food insecure	1	CCHS	Binary
	Visible minority status	White; Visible Minority	1	CCHS	Binary
	Marital status	Married or Common Law; Single or Never Married; Widowed, Separated, or Divorced	2	CCHS	Categorical
	Employment status	Employed (worked last week); Employed (absent last week); Unemployed (last week)	2	CCHS	Categorical
Health Behaviours	Alcohol consumption	Regular drinker: ≥1x/week; Occasional drinker: <1x/week but > 1x/year; Non-drinker: none in past year	2	CCHS	Ordinal
	Cigarette smoking	Never smoker; Former smoker; Current smoker	2	CCHS	Categorical
	Fruit and vegetable intake	< 3 servings/day; 3–5 servings/day; >5 servings/day	2	CCHS	Ordinal
	Physical activity	Inactive; Moderately active; Active	2	CCHS	Ordinal
Chronic Conditions	Pre-pregnancy diabetes	Yes/No	1	CCHS	Binary
	Hypertension	Yes/No	1	CCHS	Binary
	Asthma	Yes/No	1	CCHS	Binary
	Arthritis	Yes/No	1	CCHS	Binary
	Back problems	Yes/No	1	CCHS	Binary
	Migraines	Yes/No	1	CCHS	Binary
	Intestinal ulcers	Yes/No	1	CCHS	Binary
	Urinary incontinence	Yes/No	1	CCHS	Binary
	Bowel disease	Yes/No	1	CCHS	Binary
	Multimorbidity	≥ 2 chronic diseases; <2 chronic diseases	1	CCHS	Binary
Psychosocial Stress & Perception	Life stress	Not at all/Not very; A bit; Quite a bit/Extreme stress	2	CCHS	Ordinal
	Community belonging	Very weak/Somewhat weak; Somewhat strong/Very strong	1	CCHS	Binary
	Life satisfaction	Satisfied; Neither; Dissatisfied	2	CCHS	Ordinal
	Self-rated health	Poor/Fair; Good; Very good/Excellent	2	CCHS	Ordinal
	Self-rated mental health	Poor/Fair; Good; Very good/Excellent	2	CCHS	Ordinal
Reproductive & Preconception Health	BMI	Continuous	4	CCHS	Continuous
	Parity (last 5 years)	Yes/No	1	CCHS	Binary
	Folic acid use	Yes/No	1	CCHS	Binary
	History of spontaneous abortion	None; 1 previous miscarriage; >1 previous miscarriage.	2	DAD	Ordinal
	History of preterm delivery	None; 1–3 previous preterm births; ≥4 previous preterm births.	2	DAD	Ordinal
	History of live birth	None; 1–3 previous live births; ≥4 previous live births.	2	DAD	Ordinal

Vertical Axis: Multilevel Determinants of Health From biological factors to broader systemic and environmental influences					
Biomedical & Health Services	Access to regular doctor (past 12 months)	Yes/No	1	CCHS	Binary
	Ever had a pap smear	Yes/No	1	CCHS	Binary
	Flu shot (past 12 months)	Yes/No	1	CCHS	Binary
	Ever had a mental health consult	Yes/No	1	CCHS	Binary
Built Environment	Urbanicity	Urban residence; Rural residence	1	CCHS	Binary
	Intersection density	Number of three-way or greater intersections per km²	4	Can-ALE	Continuous
	Dwelling density	Number of residential units per km²	4	Can-ALE	Continuous
	Transit stops	Density of public transit stops within a given area	4	Can-ALE	Continuous
	Active living environment index	Composite measure (intersection density, dwelling density, proximity to destinations, access to public transit), standardized and categorized into 5 classes (C1 to C5)	4	Can-ALE	Ordinal
Environmental Exposures	PM2.5 concentrations	Annual fine particulate matter (µg/m³) from satellite and modeled data, calibrated to monitoring stations	4	CANUE	Continuous
	NO₂ concentrations	Annual nitrogen dioxide (ppb) from land-use regression models	4	CANUE	Continuous
	Ozone (O3)	Annual average ground-level ozone (ppb) from atmospheric models and monitoring stations	4	CANUE	Continuous
	Greenness Index (GRLAN)	Normalized Difference Vegetation Index (–1 to 1), higher values indicate more surrounding green space	4	CANUE	Continuous
Area-level Social Determinants	Neighbourhood deprivation	Quintile 1 (least deprived) to Quintile 5 (most deprived)	4	CAN-Marg	Ordinal
	Ethnic concentration	Quintile 1 (least concentrated) to Quintile 5 (most concentrated)	4	CAN-Marg	Ordinal
	Residential instability	Quintile 1 (least stable) to Quintile 5 (most stable)	4	CAN-Marg	Ordinal
	Dependency	Quintile 1 (least dependent) to Quintile 5 (most dependent)	4	CAN-Marg	Ordinal
	Neighbourhood income	Quintile 1 (lowest) to Quintile 5 (highest)	4	Census	Ordinal

*Abbreviations: CCHS *Canadian Community Health Survey, *DAD *Discharge Abstract Database, *CANUE *Canadian Urban Environmental Health Research Consortium, *Can-ALE *Canadian Active Living Environments database, *CAN-Marg * Canadian Marginalization Index, *Census* Canadian Census

^1^ Variables are organized according to Kramer et al. health equity for maternal health framework, [14] with the horizontal axis capturing accumulated social and health exposures and the vertical axis representing multilevel determinants of health

^2^ Degrees of freedom (df) reflect how each variable is modeled in the prediction model. For categorical and ordinal variables, df = k − 1, where k is the number of categories. For continuous variables modeled using restricted cubic splines, df = number of spline terms

^3^ Scale refers to how the variable is treated analytically: Continuous (numeric values), Ordinal (ordered categories), Categorical (unordered categories), or Binary (two categories)

### Outcomes

The primary outcome for PregPoRT is a composite measure of APOs, defined as the occurrence of at least one of the following conditions during delivery hospitalization: gestational diabetes mellitus, preeclampsia, or placental abruption. Gestational diabetes is a common pregnancy complication associated with elevated risks of caesarean delivery, macrosomia, neonatal hypoglycemia, and future type 2 diabetes for both mother and child [33]. Preeclampsia, a hypertensive disorder of pregnancy, is a major cause of maternal morbidity and is linked to preterm birth, intrauterine growth restriction, and long-term cardiovascular risk [34]. Placental abruption, though less prevalent, is associated with severe maternal hemorrhage, fetal mortality, and other obstetric complications [35].

These outcomes were selected based on their substantial contributions to maternal and neonatal morbidity, their frequent use in epidemiologic and clinical research, and the availability of corresponding diagnostic codes in the DAD. Also, these outcomes have established associations with modifiable risk factors, positioning them as actionable targets for public health intervention. For example, gestational diabetes has been linked to food insecurity [4, 36], placental abruption to inadequate prenatal care [35], and preeclampsia to chronic stress [36]. These conditions represent meaningful endpoints for population surveillance and prevention efforts.

Preeclampsia and eclampsia were defined by ICD-9 codes 642.3–642.7 and ICD-10 codes O14x, O15x, and O11x. Gestational diabetes was defined by ICD-9 codes 648.0 and 648.8 and ICD-10 codes O24.4, O24.8, and O24.9. Placental abruption was defined by ICD-9 code 641.2 and ICD-10 code O45. Diagnostic codes for gestational diabetes [37] and preeclampsia [38–40] have been validated in administrative data, demonstrating high specificity and moderate-to-high sensitivity, which supports their use in population-level prediction models. Although placental abruption codes have not undergone formal validation, similar placental complication codes have shown good performance in administrative datasets, suggesting acceptable reliability [41].

The use of a composite outcome increases the number of outcome events available for model development, acknowledges overlapping risk pathways, and reflects the multifactorial nature of pregnancy complications. Nonetheless, each APO will also be examined descriptively to illustrate their individual contribution to the composite outcome.

### Sample size

We used the pmsampsize package in R to calculate the minimum sample size required for prediction model development. This package implements methods proposed by Riley et al. (2019) to ensure that models are developed with sufficient precision, minimizing the risk of overfitting while allowing for reliable estimation of regression coefficients [42]. For our binary outcome, we specified an outcome prevalence of 7.6% based on preliminary analysis of our data, a conservative Nagelkerke R² of 0.20 to reflect the expected proportion of variance explained, and a shrinkage factor of 0.90 to limit optimism in predictor effect estimates. We included 118 candidate regression coefficients, reflecting the full set of planned predictor variables and their parameterizations (Table 1). Based on these inputs, the minimum required sample size was 12,196, corresponding to approximately 927 events and an events-per-parameter ratio of 7.9. Our available sample of 13,360 deliveries, therefore, exceeds this threshold.

### Analysis plan

PregPoRT will be developed in accordance with best practices for prognostic modeling, guided by the methodological recommendations of Steyerberg and Harrell [25, 26]. The analytic plan was finalized after the study cohort was created but prior to any model fitting or examination of descriptive statistics on predictor–outcome relationships. Key features of this approach include the full pre-specification of candidate predictors and the use of flexible modeling functions for continuous variables to capture potential nonlinear effects.

Aligned with recent calls for improved rigor and transparency in the design and reporting of prediction models, this protocol aims to strengthen the robustness of the PregPoRT model, enhance reproducibility, and minimize the risk of overfitting and overly optimistic performance estimates. All data cleaning, coding of predictors, and model development will be performed in R, using packages including Hmisc [43] and rms [44] for model specification and validation and survey [45] to account for the complex sampling design of the CCHS, which incorporates stratification, multistage cluster sampling, and unequal selection probabilities. The protocol was developed in accordance with the TRIPOD statement [24], which will also guide the reporting of PregPoRT to ensure adherence to established standards for multivariable prediction models.

#### Coding and cleaning of predictor variables

All data cleaning and coding of predictor variables will be completed prior to examining exposure–outcome relationships to prevent data-driven bias. Descriptive statistics and graphical methods (e.g., boxplots, histograms) will be used to assess the distribution of continuous variables and identify outliers. Continuous predictors will be modeled using restricted cubic splines to capture nonlinear relationships, and mild winsorization at the 99.5th percentile will be applied only to address extreme outliers and improve numerical stability, consistent with previous population-based risk tools [18, 46]. Modeling decisions will also be informed by prior population-based risk prediction models, such as the Premature Mortality Population Risk Tool (PreMPoRT) [47] and the Chronic Disease Population Risk Tool (CDPoRT) [48], which similarly employed pre-specification of predictors, graphical assessment of distributions, and spline-based modeling approaches to ensure robustness and interpretability. Following these approaches, all candidate predictors and their definitions were pre-specified (Table 1) to minimize the risk of overfitting and ensure methodological transparency and consistency across analyses.

#### Missing data

Patterns and correlates of missingness will be examined to evaluate the plausibility of the Missing at Random (MAR) assumption before applying multiple imputation [49]. If evidence suggests that the assumption is violated, alternative strategies such as complete case or mode imputation will be explored in sensitivity analyses.

Multiple imputation by chained equations (MICE) will be performed using the mice package [50, 51] in R, incorporating all predictor variables, the outcome, and auxiliary variables. Individuals with missing outcome data will be excluded from model development. The outcome variable will be included as an auxiliary variable in the imputation models to preserve relationships among predictors but will not be imputed.

Imputation will proceed in three stages. First, within-cycle imputations will be conducted separately for each CCHS cycle to respect cycle-specific variable structures. Second, for variables that are completely missing in one or more cycles, a between-cycle imputation will be performed using information from cycles where the variable is available. Third, environmental variables (i.e., CAN-Marg, CANUE, and CanALE) will be imputed separately to account for their distinct area-level nature and anticipated missingness patterns. Five imputed datasets will be created, and results will be pooled using Rubin’s rules [52]. This approach is informed by recent evidence showing that multiple imputation provides less biased parameter estimates compared to complete case or mode imputation for predictive models, particularly for variables with higher levels of missingness, while overall model performance measures remain similar across methods [53]. To assess the robustness of this approach, a sensitivity analysis will be conducted using a subset of the data with a larger number of imputations (e.g., 20) to confirm that model coefficients and performance metrics are consistent with those derived from the primary five-imputation models.

#### Model specification

A Weibull accelerated failure time (AFT) model will initially be fit using all candidate predictors identified through the conceptual framework (Table 1). The Weibull distribution was selected based on the characteristics of the outcome and underlying data: APOs are time-to-event processes occurring within a defined gestational period, with risk that generally increases or remains non-decreasing as pregnancy progresses. Preliminary descriptive analyses of our dataset support this pattern, demonstrating an approximately monotonically increasing hazard over gestation. The Weibull model accommodates such monotonic or near-monotonic hazard patterns while allowing direct estimation of absolute risk, making it well suited for population-based prediction modelling of pregnancy outcomes. Additionally, prior studies have shown strong performance of Weibull models in population-based prediction settings, including diabetes [18], chronic disease [54], premature mortality [55], and maternal outcomes [56, 57].

Continuous variables will be modeled using restricted cubic splines with piecewise cubic functions smoothed at knot placements based on Harrell’s percentile recommendations, allowing for flexible modeling of nonlinear associations [25]. Variables will be mean-centered to improve interpretability of coefficients and facilitate recalibration in new populations. For future external validation, recalibration will follow recommended approaches by Sim and colleagues (2016) [58]. Where outcome incidence differences between populations, recalibration-in-the-large will be used to adjust the model intercept. When the calibration slope deviates from unity, logistic recalibration will be applied to estimate an updated intercept and slope based on the linear predictor from the original model. These approaches allow for efficient model transportability while preserving the effects of predictors.

Sensitivity analyses will compare spline-based models to simpler specifications treating predictors as linear continuous terms. The functional form that yields better overall predictive performance, discrimination, calibration, and model fit (evaluated using measures such as AIC and BIC) will be chosen for the final model. To improve model stability and convergence, variables showing near-zero variability or duplication (e.g., redundant indicators of the same construct) will be removed prior to model fitting. This pragmatic pre-screening step is intended solely to ensure computational feasibility and does not serve as a data-driven variable selection method.

Variable selection will be conducted using the least absolute shrinkage and selection operator (LASSO), a penalized regression approach that balances model complexity with predictive performance [59]. All candidate predictors will be pre-specified based on the conceptual health equity–informed framework [14] and prior evidence; therefore, LASSO will be applied as a regularization technique within this predefined set rather than as a data-driven selection procedure. This approach follows guidance from Harrell [25] where penalization is used to address multicollinearity and improve coefficient stability, not to determine variable inclusion or exclusion. The optimal tuning parameter (lambda) will be identified via 10-fold cross-validation. Where LASSO retains only one level of a categorical variable, we will evaluate whether collapsing categories or retaining the full variable yields better discrimination and calibration.

Interaction terms, particularly with maternal age and key sociodemographic factors (e.g., household income, immigration status, visible minority status), will be evaluated for inclusion by assessing their contribution to model performance (e.g., discrimination, calibration). Model specification decisions will be documented transparently to align with TRIPOD guidelines and facilitate replication.

#### Model estimation

Survival-free time to diagnosis of an APO will be modeled using a Weibull AFT model as specified above. Maternal death, although rare, will be treated as a competing risk and addressed through censoring at the time of death to avoid bias in APO risk estimation.

To confirm that the Weibull model is appropriate for these data, we will assess the adequacy of the Weibull distribution and the AFT assumption using multiple diagnostics [60]. First, we will estimate the Weibull shape (scale) parameter and evaluate whether it significantly differs from 1, which would indicate a deviation from the exponential distribution and justify the use of a Weibull model. We will then examine Cox-Snell residuals and plots of log-transformed survival time against the linear predictor to assess linearity and homoscedasticity, which are key assumptions of the AFT framework. Model fit will further be evaluated by comparing alternative parametric specifications (e.g., exponential, log-logistic) using Akaike Information Criterion (AIC) and graphical diagnostics.

To ensure representativeness of the Canadian population and account for the complex sampling design of the CCHS, Statistics Canada survey weights will be applied throughout the analysis [28].

#### Model validation

**Fig. 1:**
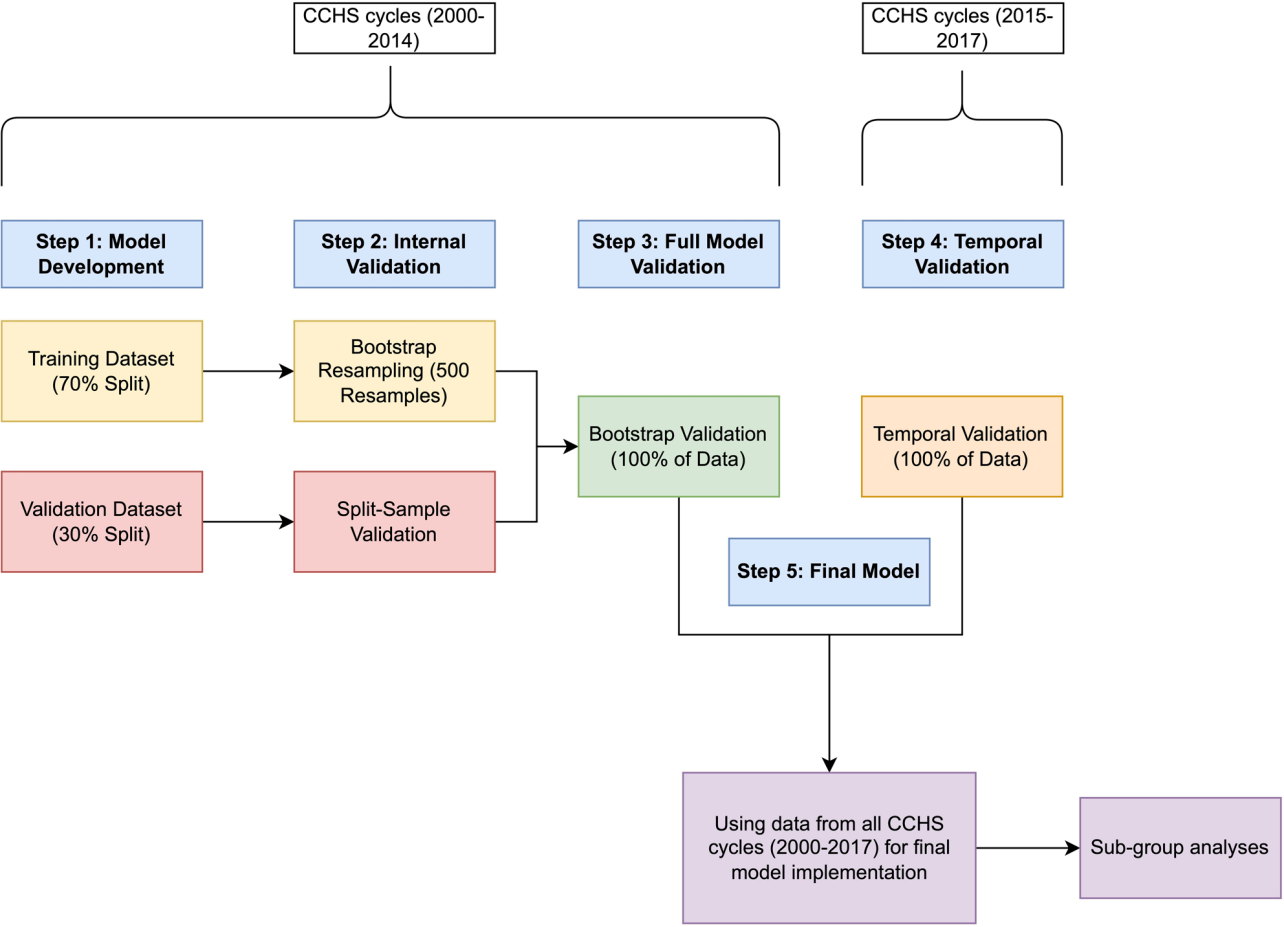
Steps Used in the Development and Validation of the Adverse Pregnancy Outcomes Population Risk Tool (PregPoRT). PregPoRT will be developed using a 70% random sample from the first seven CCHS cycles (2000/01 to 2013/14) (Step 1). Internal validation will be performed on this 70% split using bootstrap analysis with 500 resamples (Step 2a), while the remaining 30% will be used for split-sample validation (Step 2b). After adjusting for overfitting with a shrinkage factor, the entire dataset (100%) will be combined to estimate the final model, with further internal validation via bootstrapping (Step 3). Temporal validation will follow using the 2015/16 and 2017/18 CCHS cycles to confirm predictive accuracy post-survey redesign (Step 4). Finally, the model will be validated across all CCHS cycles (2000 to 2017) and assessed for performance consistency across diverse demographics (Step 5)

Figure 1 shows a visual representation of the steps used in the development and validation of PregPoRT. The model will initially be developed using a 70% random sample of respondents from the first seven CCHS cycles (2000/01 to 2013/14) (Fig. 1, Step 1). Internal validation will be performed on this 70% development sample using bootstrap resampling with 500 iterations, as recommended by Deng et al. [61], who found this number provides stable confidence intervals without inflating computation time (Fig. 1, Step 2a). For each bootstrap iteration, a sample of the same size as the development dataset will be drawn with replacement, the model will be refit, and its performance will be evaluated using metrics such as Nagelkerke’s R² and the C-statistic. Model performance in each bootstrap resample will then be compared to that in the original sample to estimate optimism. Average optimism across resamples will be used to adjust performance metrics and, if necessary, to derive a uniform shrinkage factor that proportionally scales model coefficients to reduce overfitting.

As an additional check, the remaining 30% of the data will be used for split-sample validation (Fig. 1, Step 2b). Finally, to avoid the inefficiencies of split-sample modeling, the entire dataset (100%) will be combined to estimate the final PregPoRT model, with internal validation again conducted via bootstrap resampling to obtain bias-corrected performance estimates and robust standard errors (Fig. 1, Step 3).

Following this, a temporal validation (Fig. 1, Step 4) will be conducted on the two most recent CCHS cycles: 2015/16 and 2017/18. These cycles were chosen due to the significant redesign of the CCHS in 2015, which included updates to the sampling methodology, health-related content, and target population [28]. Validating the model with these data will confirm its predictive accuracy within the context of the survey’s updated design. Upon successful validation, we will merge data from all CCHS cycles (2000 to 2017) to estimate the final model application (Fig. 1, Step 5). We will also assess model performance across various geographic locations, education levels, income brackets, and immigration status to ensure consistent accuracy across diverse demographics. If substantial disparities in performance are identified, we will explore subgroup-specific recalibration (e.g., adjusting intercepts or calibration slopes) and will report subgroup metrics transparently in accordance with TRIPOD, even if full recalibration is not pursued.

#### Performance assessment

Overall predictive accuracy will be evaluated using Nagelkerke’s R² and the Integrated Brier Score. Nagelkerke’s R² provides an index of explained variation and overall model fit, while the Integrated Brier Score summarizes both discrimination and calibration to capture the model’s overall accuracy [62]. Discrimination—the ability of the model to distinguish between individuals who experience an APO and those who do not—will be quantified using Harrell’s concordance statistic (C-statistic) [63].

Calibration, or the agreement between predicted and observed risks, will be evaluated following the recommendations of Van Calster et al. (2019) and Austin et al. (2020) [64]. Calibration will be assessed at several levels: (1) *calibration-in-the-large*, by comparing the average predicted risk to the observed event rate; (2) *weak calibration*, using the calibration intercept (target = 0) and slope (target = 1) estimated by regressing observed outcomes on the model’s linear predictor; and (3) *moderate calibration*, through flexible calibration curves (e.g., loess or spline-smoothed plots of observed versus predicted risk). The overall extent of miscalibration will be quantified using the Integrated Calibration Index (ICI), which summarizes the average absolute difference between predicted and observed risks across all risk levels. A curve close to the 45-degree line will indicate good agreement. When miscalibration is observed, recalibration-in-the-large (adjusting the intercept) or logistic recalibration (adjusting intercept and slope) will be applied as recommended by Sim and colleagues [58].

Model performance will be summarized using both mean and median values across the imputed datasets, providing robust estimates less sensitive to outliers [65].

#### Model presentation

The final PregPoRT model will be presented with estimated coefficients, hazard ratios, baseline survival function, and 95% confidence intervals, enabling independent external validation and implementation. To illustrate the model’s predictive capacity, we will display the distribution of APO risk across the cohort and examine subgroup performance across key sociodemographic groups. Because the primary aim is to generate population-based predictions, we will also make the model coefficients publicly available for replication and application in new settings.

PregPoRT is intended for population health surveillance and planning rather than individual clinical decision-making and therefore is not classified as a medical device under current regulatory definitions. Should future iterations be adapted for clinical use, appropriate regulatory pathways will be evaluated.

To support broad uptake, we will provide open-access R code and documentation via GitHub. Model dissemination and knowledge translation will be guided by the Population Health Planning Knowledge-to-Action Model, which we have successfully used in previous projects to deploy population risk tools in real-world public health settings [66, 67]. In prior work, this approach, combined with tailored training and knowledge brokering strategies, facilitated integration of the Diabetes Population Risk Tool (DPoRT) into decision-making processes at multiple health organizations, including Peel Public Health, resulting in changes to skills, knowledge, and organizational practices [68]. Building on this experience, once PregPoRT is validated, we will collaborate with local public health units to deliver training workshops demonstrating how the tool can be applied to identify high-risk groups, inform prevention strategies, and support equitable policy and program planning.

Importantly, use of PregPoRT should be accompanied by evaluations of the benefits achieved, ideally supported by high-quality evidence on the effectiveness of proposed interventions. Comparable work with the Diabetes Population Risk Tool (DPoRT) demonstrated that impact evaluations are essential to understanding how prediction tools translate into practice [67, 69]. In evaluations of DPoRT, integration into regional planning processes was associated with improved targeting of prevention strategies and measurable changes in knowledge, skills, and organizational practices within public health units [67]. Building on this precedent, future evaluations of PregPoRT could examine whether adoption by public health units leads to improved identification of high-risk groups, better alignment of prenatal care resources, or reductions in adverse pregnancy outcomes over time. Situating PregPoRT within this broader evidence-to-action cycle will help ensure the tool generates both immediate practical value and long-term improvements in maternal and fetal health.

## Discussion

APOs are key indicators of health system performance, with important implications for both short- and long-term population health. As such, they are increasingly recognized as meaningful markers in health system evaluation and improvement efforts. Despite this, current risk prediction tools for APOs have largely been developed for use in clinical settings and are designed to guide individual-level care rather than inform public health action.^60^ These tools often rely on clinical or biological inputs such as biomarkers, family history, or detailed hospital records, data that are not routinely collected at the population level and are inaccessible to public health decision-makers.

Despite maternal health being a top public health priority, currently, there is no streamlined or widely adopted approach for health system planners to estimate the incidence of APOs at a population level. A tool that captures risk based on routinely available, population-based data, particularly one that integrates social, behavioral, and environmental risk factors, can support public health units and healthcare planners in identifying at-risk groups, targeting interventions, and tracking improvements over time. The PregPoRT model is designed to fill this gap by using linked population survey, administrative, and environmental data to generate tailored, population-level risk estimates of APOs.

Importantly, PregPoRT focuses on predictors that are both modifiable and routinely collected in population health surveillance systems, supporting its application in public health planning, health equity monitoring, and resource allocation. While this model is developed to estimate the risk of a composite APO outcome (gestational diabetes, preeclampsia, and placental abruption), its flexible design allows for future extensions. Upcoming models could consider a broader range of perinatal outcomes (e.g., preterm birth, stillbirth, or severe maternal morbidity) to represent additional critical indicators of maternal and fetal well-being.

By focusing on population-level risk prediction, PregPoRT represents a novel and scalable approach to improving maternal health through prevention, planning, and policy action. As maternal health continues to be shaped by social and structural factors, tools like PregPoRT can help shift the focus of perinatal care and policy upstream, toward interventions that are proactive, equitable, and grounded in the lived realities of pregnant individuals.

### Limitations

This research has several limitations. First, our study cohort is restricted to CCHS respondents who provided consent to link their data with the DAD. While the consent rate is high (i.e., > 80% of respondents) there remains the potential for systematic differences between those who consented and those who did not, as well as for survey non-response bias [47]. To partially address these risks, we will apply Statistics Canada’s survey weights, which adjust for differential selection probabilities and survey non-response [28]. However, these weights do not account for potential differences between those who consented to linkage and those who did not, which remains an important limitation.

Second, the DAD captures only hospital-based deliveries, excluding those that occur at home or in birthing centers. These out-of-hospital deliveries typically involve lower-risk pregnancies, and their exclusion may slightly inflate the risk profile observed in our cohort. Nevertheless, because approximately 98% of Canadian births occur in hospitals [70], this limitation is unlikely to meaningfully affect the representativeness of our findings.

Third, our analytic cohort includes only deliveries resulting in a live birth or stillbirth recorded in the DAD, omitting miscarriages and early pregnancy losses. This exclusion may underestimate the full burden of APOs in the population [46]. To address this limitation, we plan to conduct a sensitivity analysis among individuals in the CCHS who reported being pregnant but did not appear in the DAD, which may provide insight into the extent of under-ascertainment and its potential impact on model predictions.

Fourth, the study cohort spans nearly two decades (2000–2017), during which healthcare delivery, prenatal screening, and management practices may have evolved. Such temporal changes could influence the distribution of exposures and the incidence of APOs. We will explore temporal trends descriptively and, where necessary, include calendar time as a covariate in the prediction model to account for these secular shifts. In addition, because the most recent data end in 2017, the model does not capture more recent changes in obstetrical care, healthcare access, or population health that have occurred in the past eight years, particularly those related to the COVID-19 pandemic.

Fifth, the CCHS relies on self-reported information for many sociodemographic, behavioral, and health-related variables. This may introduce recall bias or social desirability bias, particularly for sensitive behaviors such as alcohol or tobacco use. Despite this limitation, CCHS data have been successfully employed in prior population-based prediction models across multiple health domains [18, 46, 54, 55, 71], and their broad availability and coverage remain significant strengths for public health applications.

Finally, our data exclude certain populations due to survey design and data-sharing restrictions, most notably on-reserve Indigenous peoples, institutionalized individuals, and residents of Quebec [28]. This exclusion limits the national generalizability of PregPoRT, particularly given evidence of higher APO rates among Indigenous populations in Canada [72]. At present, there are no comparable linked survey–administrative datasets that would enable validation of PregPoRT in these groups. Addressing this gap will require dedicated data collection efforts in partnership with Indigenous communities and jurisdictions not currently represented, which should be a priority for future research.

## Conclusions

We anticipate that PregPort will be a valuable addition to the resources available to regional, provincial, and national decision-makers, strengthening maternal health surveillance, informing pregnancy-related research, and guiding public health planning and policy efforts aimed at improving the health of mothers and children.

## Data Availability

The data are held within Statistics Canada’s Research Data Centre (RDC) network and are not publicly available due to privacy and governance requirements under the Statistics Act. Access is limited to approved researchers with peer-reviewed projects authorized by Statistics Canada. Applications are submitted through the Microdata Access Portal; approved researchers work within secure RDC facilities or, where available, via remote desktop. All analyses occur in the secure environment, and outputs undergo Statistics Canada’s vetting before release. Further information is available at https://www.statcan.gc.ca/en/microdata/data-centres/access. Environmental data were provided by the Canadian Urban Environmental Health Research Consortium (CANUE) under a study-specific Data Sharing and Use Agreement and cannot be redistributed; access may be requested directly from CANUE.
